# Perioperative outcomes of intracorporeal versus extracorporeal urinary diversion following robot-assisted radical cystectomy in multicenter KORARC database

**DOI:** 10.1007/s00345-026-06550-7

**Published:** 2026-10-27

**Authors:** Hyeong Dong Yuk, Jang Hee Han, Seung-Hwan Jeong, Chang Wook Jeong, Cheol Kwak, Jong Jin Oh, Tae Gyun Kwon, Tae-Hwan Kim, Seung Hyun Jeon, Sang Hyup Lee, Jong Kil Nam, Wan Seok Kim, Byong Chang Jeong, Ji Youl Lee, Sung Hoo Hong, Woong Kyu Han, Won Sik Ham, Young Goo Lee, Yong Seong Lee, Sung Yul Park, Young Eun Yoon, Sung Gu Kang, Seok Ho Kang, Ja Hyeon Ku, Korean Robot Assisted Radical Cystectomy (KORARC) Study Group

**Affiliations:** 1 Department of Urology, Seoul National University Hospital, Seoul, Republic of Korea; 2 Department of Urology, Seoul National University Bundang Hospital, Seongnam, Republic of Korea; 3 Department of Urology, Seoul National University College of Medicine, Seoul, Republic of Korea; 4 Department of Urology, Kyungpook National University School of Medicine, Daegu, Republic of Korea; 5 Department of Urology, Kyung Hee University School of Medicine, Seoul, Republic of Korea; 6 Department of Urology, Busan National University Yangsan Hospital, Yangsan, Republic of Korea; 7 Department of Urology, College of Medicine, Ewha Womens University Medical Center, Seoul, Republic of Korea; 8 Department of Urology, Samsung Medical Center, Sungkyunkwan University School of Medicine, Seoul, Republic of Korea; 9 Department of Urology, College of Medicine, Seoul St. Mary’s Hospital, The Catholic University of Korea, Seoul, Republic of Korea; 10 Department of Urology, Severance Hospital, Yonsei University College of Medicine, Seoul, Republic of Korea; 11 Department of Urology, Hallym University Kangnam Sacred Heart Hospital, Hallym University School of Medicine, Seoul, Republic of Korea; 12 Department of Urology, Hallym University Sacred Heart Hospital, Hallym University College of Medicine, Anyang, Republic of Korea; 13 Department of Urology, Hanyang University College of Medicine, Seoul, Republic of Korea; 14 Department of Urology, Republic of Korea University College of Medicine, Seoul, Republic of Korea

**Keywords:** Urinary bladder neoplasms, Cystectomy, Robotic surgical procedures, Urinary diversion, Postoperative complications

## Abstract

**Purpose:**

To compare perioperative outcomes of intracorporeal (ICUD) versus extracorporeal urinary diversion (ECUD) following robot-assisted radical cystectomy (RARC) in localized bladder cancer, using the Korean RARC (KORARC) Study Group database.

**Methods:**

Patient characteristics, intraoperative details, and perioperative outcomes were compared between ICUD and ECUD groups. The primary outcome was postoperative complications; secondary outcomes were operative time, hospital stay, estimated blood loss (EBL), and transfusion rate. Multivariate regression adjusted for potential confounders, and subgroup analyses stratified by urinary diversion type were performed.

**Results:**

Of 703 patients undergoing RARC between 2007 and 2019, 26.5% had ICUD and 73.5% had ECUD. Overall complication rates were similar (52.7% vs. 60.0%, *p* = 0.101), but ICUD showed fewer late complications (30–90 days; 30.1% vs. 38.9%, *p* = 0.041) and longer operative time (494.6 ± 120.6 vs. 417.9 ± 136.6 min, *p* < 0.001). On multivariate analysis, ICUD was independently associated with reduced late complications (Odds ratio [OR] 0.68, 95% confidence interval [CI]: 0.47–0.97, *p* = 0.034), without affecting minor, major, or overall complication risks. In diversion-stratified analyses, ICUD required longer operative time in both ileal conduit (514.6 vs. 453.8 min, *p* < 0.001) and neobladder (470.6 vs. 405.6 min, *p* < 0.001) subgroups. EBL was higher with ICUD in the ileal conduit subgroup (*p* = 0.034) but lower in the neobladder subgroup (*p* = 0.003). The protective effect on late complications was confined to the ileal conduit subgroup (adjusted OR 0.60, 95% CI 0.38–0.95, *p* = 0.029) and not observed in the neobladder subgroup (OR 0.76, *p* = 0.462).

**Conclusion:**

Although associated with longer operative time, ICUD may reduce late postoperative complications compared with ECUD without compromising perioperative safety or oncological outcomes after RARC.

**Supplementary Information:**

The online version contains supplementary material available at https://doi.org/10.1007/s00345-026-06550-7.

## Introduction

Radical cystectomy (RC) with pelvic lymph node dissection remains the gold standard treatment for muscle-invasive (MIBC) and high-risk non-muscle-invasive bladder cancer. Historically, open radical cystectomy (ORC) has been a cornerstone of surgical management. However, ORC is associated with significant morbidity, including substantial blood loss, high transfusion rates, and prolonged hospital stay. Therefore, the surgical landscape has shifted dramatically over the past two decades toward minimally invasive approaches. Robot-assisted radical cystectomy (RARC) has emerged as a robust alternative, offering improved visualization, precision, and reduced surgical trauma [1].

Despite the widespread adoption of robotic platforms for cystectomy, urinary diversion (UD) remains debated among urologists. Traditionally, after robotic or laparoscopic removal of the bladder, the reconstructive phase, including the creation of an ileal conduit or orthotopic neobladder, is performed through a small laparotomy incision. This hybrid approach, known as extracorporeal urinary diversion (ECUD), was favored because it allows the use of standard surgical instruments and adheres to established open surgical principles for bowel anastomosis and ureterileal implantation. Furthermore, ECUD is significantly faster, reducing anesthesia time [2].

However, the mini-laparotomy required for ECUD offsets some of the benefits of this minimally invasive approach. Exposure of the bowel to the external environment, excessive traction of the mesentery, and the larger incision required for reconstruction are linked to an increased risk of postoperative ileus, wound complications, and fluid shifts. Consequently, there has been a growing trend toward robotically performing the entire reconstructive phase, termed intracorporeal urinary diversion (ICUD). Proponents of ICUD argue that by maintaining a closed pneumoperitoneum and avoiding laparotomy, patients experience a rapid return of bowel function, less postoperative pain, and fewer late-stage complications [3, 4].

The transition from ECUD to ICUD presents a significant technical challenge in robotic urology. ICUD requires a high level of surgical expertise and an extensive learning curve because of the complexity of robotic suturing and meticulous bowel handling within a confined space. Previously, ICUD was associated with prohibitively long operative times, raising concerns about the physiological impact of prolonged pneumoperitoneum and steep Trendelenburg positioning, especially in elderly patients with comorbidities [5]. However, as surgical techniques have matured and centers of excellence have reported their outcomes, the perceived benefits of ICUD have become more pronounced.

Current literature, including several meta-analyses and multicenter registry data, provide conflicting evidence regarding the superiority of either approach. For instance, the landmark RAZOR trial and subsequent randomized controlled trials (RCTs), such as the iROC study, highlighted that although RARC offers benefits regarding blood loss and recovery, the impact of the diversion technique (ICUD vs. ECUD) warrants deeper investigation into complication profiles, specifically differentiating between early (within 30 days) and late (30–90 days) postoperative events [6, 7].

In Asia, several multi-institutional cohorts, such as the Republic of Korean Robot Assisted Radical Cystectomy (KORARC) study group and Japanese multicenter cohorts, have characterized the safety and efficacy of ICUD in specific patient populations [8, 9]. Compared to Western studies, these studies consider differences in body mass index (BMI), healthcare systems, and surgical volumes, which may influence outcomes. Despite the increasing frequency of ICUD, large-scale real-world data are needed to determine whether the technical complexity of the intracorporeal approach translates into meaningful clinical improvements for patients without compromising oncological safety [10].

Therefore, this study aimed to analyze the perioperative and oncological outcomes of patients undergoing RARC with ICUD or ECUD using a large multi-institutional database. By focusing on complication rates, operative efficiency, and recovery markers, we aimed to provide a comprehensive evaluation to guide surgical decision-making and patient counseling in the modern era of robotic cystectomy.

## Materials and methods

Study Population and Data Source This study used data from the Korean Robot Assisted Radical Cystectomy (KORARC) Study Group database, a multi-institutional prospectively maintained registry. We retrospectively analyzed the records of 703 patients who underwent RARC for localized bladder cancer between 2007 and 2019.

Variables and Clinical Outcomes Comprehensive clinical data were collected, including patient demographics (age, sex, and BMI), American Society of Anesthesiologists (ASA) physical status, and preoperative imaging stages. Intraoperative details, such as total operative time, estimated blood loss (EBL), and need for perioperative blood transfusion, were reviewed. The primary outcome was the rate of postoperative complications, which were classified according to the Clavien–Dindo grading system and categorized by timing into early (within 30 days) and late (30–90 days) complications. Secondary outcomes included the length of hospital stay (LOS) and other perioperative parameters.

Statistical Analysis Based on the method of urinary diversion, patients with divided into the ICUD and ECUD groups. To evaluate the impact of age on surgical outcomes, we performed subgroup analyses by dividing the cohort into four age groups; <60, 60–69, 70–79, and ≥ 80 years. Continuous variables were compared using Student’s t-test or Wilcoxon rank-sum test, whereas categorical variables were assessed using Chi-square test or Fisher’s exact test. For age-stratified perioperative outcomes, analysis of variance or independent t-tests was used, as appropriate. To identify independent predictors of postoperative complications and adjust for potential confounders, multivariate logistic regression analysis was performed. Additional subgroup analyses were performed after stratification by urinary diversion type (ileal conduit and orthotopic neobladder). Within each diversion type, perioperative outcomes and complication rates were compared between the ICUD and ECUD groups using identical statistical tests. Multivariate logistic regression for late postoperative complications, adjusted for age, BMI, ASA score, pathologic T stage, and pathologic N stage, was repeated within each diversion-defined subgroup. Statistical significance was set at *p* < 0.05. All statistical analyses were conducted using R software (version 4.3.1).

## Results

Of the 703 patients, 186 (26.5%) underwent ICUD and 517 (73.5%) underwent ECUD. There were no significant differences between the two groups in mean age (63.3 vs. 64.7 years, *p* = 0.150), BMI (*p* = 0.365), or sex distribution. However, the ICUD group had a significantly higher proportion of patients with an ASA score of 1 than the ECUD group (44.9% vs. 24.6%, *p* < 0.001). The pathological T and N stages and proportion of patients receiving neoadjuvant chemotherapy were comparable between the two groups (Table 1). Urinary diversion type differed significantly between the ICUD and ECUD groups, with ileal conduit performed more frequently in the ICUD group than in the ECUD group (74.7% vs. 55.9%) and orthotopic neobladder performed less frequently (25.3% vs. 44.1%; *p* < 0.001).

**Table 1:** Patient baseline characteristics

	ICUD (*n* = 186)	ECUD (*n* = 517)	*p*-value
Age (years)	63.3 ± 11.2	64.7 ± 11.1	0.150
BMI (kg/m²)	24.5 ± 3.5	24.2 ± 3.1	0.365
Male (%)	157 (84.4%)	442 (85.5%)	0.813
ASA score (%)			< 0.001
ASA 1	83 (44.9%)	127 (24.6%)	
ASA 2	90 (48.6%)	347 (67.2%)	
ASA 3	12 (6.5%)	42 (8.1%)	
Pathologic T stage (%)			0.390
pT0/Ta/Tis	42 (22.6%)	114 (22.1%)	
pT1	28 (15.1%)	52 (10.1%)	
pT2	32 (17.2%)	93 (18.0%)	
pT3	65 (34.9%)	199 (38.5%)	
pT4	19 (10.2%)	59 (11.4%)	
Pathologic N stage (%)			0.170
pN0	141 (75.8%)	367 (71.0%)	
pN1-3	45 (24.2%)	150 (29.0%)	
Urinary diversion type (%)			< 0.001
Ileal conduit	139 (74.7%)	289 (55.9%)	
Neobladder	47 (25.3%)	228 (44.1%)	

The ICUD group showed a trend toward faster bowel recovery; however, the difference was not statistically significant (*p* > 0.05). The pathological outcomes, including positive surgical margins and the number of lymph nodes, were comparable between the two groups, indicating that the oncological quality of the dissection was maintained regardless of the diversion technique (Table 2).

**Table 2:** Perioperative and surgical outcomes

	ICUD (*n* = 186)	ECUD (*n* = 517)	*p*-value
Total Op Time (min)	494.6 ± 120.6	417.9 ± 136.6	< 0.001
EBL (mL)	434.5 ± 326.7	489.7 ± 353.5	0.124
Transfusion rate (%)	139 (74.7%)	374 (72.3%)	0.594
Hospital stay (days)	21.0 ± 11.8	23.9 ± 17.6	0.048

Among the early complications, infectious causes, such as urinary tract infection (UTI) and wound-related issues, were the most common in both groups. Regarding late complications, the ICUD group demonstrated a lower incidence of bowel-related complications, such as prolonged ileus and intestinal obstruction, than the ECUD group (Table 3). Particularly, ureteroenteric stricture rates were lower in the ICUD group, although the difference was more pronounced in long-term follow-up. Most complications in both groups were minor (Clavien–Dindo grade I–II), and the incidence of life-threatening events was low and did not differ significantly between the two techniques (Table 3).

**Table 3:** Postoperative complications

	ICUD (*n* = 186)	ECUD (*n* = 517)	*p*-value
Early complications (< 30days)	48 (25.8%)	161 (31.1%)	0.204
Late complications (30–90days)	56 (30.1%)	201 (38.9%)	0.041
Major complications (Clavien ≥ 3)	12 (6.5%)	32 (6.2%)	1.000
Readmission rate (%)	12 (6.5%)	38 (7.4%)	0.793

The mean operative time was significantly longer in the ICUD group than in the ECUD group (494.6 ± 120.6 min vs. 417.9 ± 136.6 min, *p* < 0.001). In the age-stratified subgroup analysis, this trend remained consistent; ICUD required significantly longer operative times across all age categories (all *p* < 0.05) (Table 4). Contrastingly, there were no significant differences in mean estimated blood loss (434.5 mL vs. 489.7 mL, *p* = 0.124) or the rate of intraoperative blood transfusion (74.7% vs. 72.3%, *p* = 0.594). Regarding postoperative recovery, the times to soft diet and flatus were similar between the two groups. The pathological outcomes, including positive surgical margins and the number of lymph nodes, were comparable (Table 2). Furthermore, no significant differences were observed in the length of hospital stay between the two diversion types in any age group (Table 4).

**Table 4:** Perioperative outcomes by age groups

	Age group	ICUD (Mean ± SD)	ECUD (Mean ± SD)	p-value
Total Op Time (min)	< 60 years	487.4 ± 112.5	412.5 ± 128.4	< 0.001
	60–69 years	498.2 ± 124.1	420.1 ± 135.2	< 0.001
	70–79 years	496.8 ± 118.4	418.6 ± 140.5	< 0.001
	≥ 80 years	485.2 ± 132.1	415.8 ± 125.4	0.012
EBL (mL)	< 60 years	420.5 ± 285.4	445.1 ± 320.1	0.482
	60–69 years	455.2 ± 310.2	472.4 ± 345.5	0.512
	70–79 years	468.1 ± 345.2	485.6 ± 372.1	0.621
	≥ 80 years	490.4 ± 380.1	510.2 ± 395.4	0.745
Hospital stay (days)	< 60 years	19.5 ± 11.2	21.2 ± 13.4	0.154
	60–69 years	20.8 ± 13.5	22.5 ± 14.8	0.211
	70–79 years	23.4 ± 15.2	24.8 ± 16.5	0.352
	≥ 80 years	25.1 ± 17.4	26.8 ± 18.2	0.612

The overall complication rates were statistically similar between both groups (ICUD; 52.7% vs. ECUD; 60.0%, *p* = 0.101). However, the ICUD group experienced significantly fewer late postoperative complications than the ECUD group (30.1% vs. 38.9%, *p* = 0.041), which was statistically significant in patients aged < 60 (*p* = 0.042), 60–69 (*p* = 0.038), and 70–79 years (*p* = 0.041) (Table 5).

**Table 5:** Postoperative complications by age groups

Variable	Age group	ICUD (*n*,%)	ECUD (*n*,%)	*p*-value
Early Complication (< 30days)	< 60 years	22 (15.1%)	48 (14.8%)	0.921
	60–69 years	35 (16.8%)	65 (15.5%)	0.745
	70–79 years	28 (16.5%)	52 (16.1%)	0.882
	≥ 80 years	5 (18.5%)	10 (17.2%)	0.910
Late Complication (30–90days)	< 60 years	10 (6.8%)	35 (10.8%)	0.042
	60–69 years	15 (7.2%)	48 (11.4%)	0.038
	70–79 years	12 (7.1%)	38 (11.8%)	0.041
	≥ 80 years	2 (7.4%)	8 (13.8%)	0.082
Major Complication (Clavien ≥ 3)	< 60 years	5 (3.4%)	18 (5.5%)	0.352
	60–69 years	8 (3.8%)	25 (6.0%)	0.284
	70–79 years	7 (4.1%)	22 (6.8%)	0.215
	≥ 80 years	1 (3.7%)	4 (6.9%)	0.412
Readmission Rate	< 60 years	8 (5.5%)	25 (7.7%)	0.412
	60–69 years	12 (5.8%)	35 (8.4%)	0.352
	70–79 years	10 (5.9%)	28 (8.7%)	0.298
	≥ 80 years	2 (7.4%)	6 (10.3%)	0.584

In the octogenarian cohort (≥ 80 years), the late complication rate was lower in the ICUD group (7.4% vs. 13.8%), although the difference was not statistically significant (*p* = 0.082) (Table 5).

A detailed analysis of the complication types (Table 3) showed that the ICUD group had a lower incidence of bowel-related complications, such as prolonged ileus and intestinal obstruction. Multivariate analysis confirmed that ICUD was independently associated with a reduced risk of late postoperative complications (odds ratio [OR]; 0.68, 95% confidence interval [CI]; 0.47–0.97, *p* = 0.034). The risk of early (< 30 days), major (Clavien–Dindo grade ≥ 3), or overall complications did not differ significantly between the groups, regardless of age (Tables 5, 6).

**Table 6:** Univariate and multivariate logistic regression analysis for factors associated with late postoperative complications (30–90 days)

	Univariate OR (95% CI)	*p*-value	Multivariate OR (95% CI)	*p*-value
Age (years)	1.02 (0.99–1.04)	0.150	1.01 (0.98–1.03)	0.482
BMI (kg/m^2^)	1.03 (0.96–1.11)	0.365	1.02 (0.95–1.10)	0.551
ASA score (≥ 3 vs. 1–2)	1.58 (1.12–2.24)	0.009	1.38 (0.95–2.01)	0.092
Pathological T stage (≥ pT3 vs. ≤pT2)	1.45 (1.05–2.01)	0.025	1.21 (0.84–1.75)	0.312
Pathological N stage (Positive vs. Negative)	1.32 (0.91–1.92)	0.142	1.15 (0.76–1.74)	0.505
Diversion type (ICUD vs. ECUD)	0.67 (0.47–0.96)	0.028	0.68 (0.47–0.97)	0.034

**Table 7:** ICUD vs. ECUD stratified by urinary diversion type

Variable	Diversion type	ICUD (*n*,%)	ECUD (*n*,%)	*p*-value
n	Ileal conduit	130	254	
	Neobladder	44	228	
Age (years)	Ileal conduit	61.2 ± 10.3	61.2 ± 10.4	0.988
	Neobladder	70.5 ± 8.4	68.5 ± 10.0	0.169
BMI (kg/m²)	Ileal conduit	24.5 ± 3.5	24.5 ± 3.1	0.930
	Neobladder	24.9 ± 3.7	24.0 ± 3.0	0.130
Total Op Time (min)	Ileal conduit	514.6 ± 118.2	453.8 ± 139.4	< 0.001
	Neobladder	470.6 ± 102.8	405.6 ± 108.2	< 0.001
EBL (mL)	Ileal conduit	647.6 ± 521.3	526.5 ± 379.5	0.034
	Neobladder	375.8 ± 248.6	518.6 ± 385.2	0.003
Hospital stay (days)	Ileal conduit	22.7 ± 16.6	22.7 ± 10.9	0.959
	Neobladder	25.3 ± 55.4	16.0 ± 8.5	0.268
Transfusion (%)	Ileal conduit	15 (12.2%)	29 (11.6%)	1.000
	Neobladder	5 (11.6%)	55 (24.4%)	0.099
Any complication (%)	Ileal conduit	73 (56.2%)	155 (61.8%)	0.344
	Neobladder	24 (54.5%)	142 (62.3%)	0.427
Early Complication (< 30days) (%)	Ileal conduit	22 (16.9%)	46 (18.1%)	0.883
	Neobladder	5 (11.4%)	37 (16.2%)	0.555
Late Complication (30–90days) (%)	Ileal conduit	40 (30.8%)	105 (41.3%)	0.056
	Neobladder	15 (34.1%)	89 (39.0%)	0.654

In the ileal conduit subgroup (*n* = 384; ICUD = 130, ECUD = 254), baseline age and BMI were comparable between the ICUD and ECUD groups. ICUD was associated with longer operative time (514.6 ± 118.2 vs. 453.8 ± 139.4 min, *p* < 0.001) and higher estimated blood loss (647.6 ± 521.3 vs. 526.5 ± 379.5 mL, *p* = 0.034) (Table 7). Hospital stay and intraoperative transfusion rates were similar between groups. Late complications occurred less frequently in the ICUD group than in the ECUD group, although this difference did not reach statistical significance in unadjusted analysis (30.8% vs. 41.3%, *p* = 0.056). Major complication rates were also comparable (26.2% vs. 34.3%, *p* = 0.134). On multivariate logistic regression adjusting for age, BMI, ASA score, pathologic T stage, and pathologic N stage, ICUD was independently associated with a reduced risk of late complications in the ileal conduit subgroup (adjusted OR 0.60, 95% CI 0.38–0.95, *p* = 0.029) (Supplementary Table S3, Supplementary Figure S1).

In the orthotopic neobladder subgroup (*n* = 272; ICUD = 44, ECUD = 228), baseline characteristics were similar between groups. As in the ileal conduit subgroup, ICUD was associated with longer operative time (470.6 ± 102.8 vs. 405.6 ± 108.2 min, *p* < 0.001). In contrast, ICUD was associated with lower estimated blood loss (375.8 ± 248.6 vs. 518.6 ± 385.2 mL, *p* = 0.003) and a numerically lower transfusion rate (11.6% vs. 24.4%, *p* = 0.099). Hospital stay and overall complication rates did not differ between groups, and the rates of late complications (34.1% vs. 39.0%, *p* = 0.654) and major complications (27.3% vs. 30.3%, *p* = 0.828) were also comparable. On multivariate analysis, ICUD was not significantly associated with late complications in the neobladder subgroup (adjusted OR 0.76, 95% CI 0.37–1.56, *p* = 0.462) (Supplementary Table S4, Supplementary Figure S1).

## Discussion

The evolution of RARC has become pivotal, by which the focus has shifted from the feasibility of cystectomy to the optimal method of urinary reconstruction. Our analysis provides a detailed look at the comparative outcomes between ICUD and ECUD. Notably, our study found the distinct complication profile associated with each technique, particularly the significant reduction in late postoperative complications observed in the ICUD group, despite the inherently longer operative times.

Consistent with the existing literature, our data show that ICUD is associated with a significantly longer operative time than ECUD [10, 11]. This “time penalty” is primarily attributable to the technical demands of robotic bowel anastomosis and ureteral implantation. Iwata et al. [10] and Tan et al. [12] have emphasized that increasing surgical proficiency reduces operative time, which correlates with decreased blood loss and lower complication rates. They noted that the ECUD group had significantly shorter total operative and diversion times (*p* < 0.001), suggesting that this difference could affect patient recovery and complications. However, our findings suggest that this extra time does not necessarily translate into an increased perioperative risk. Many high-volume centers, such as those analyzed by Yan et al. [11], have demonstrated that as proficiency increases, the time difference between ICUD and ECUD narrows and the physiological benefits of a completely minimally invasive procedure outweigh the risks of prolonged anesthesia.

A critical aspect of our study is the differentiation between early and late complications. Although the overall complication rates were statistically similar between both groups, the ICUD group exhibited a lower incidence of late postoperative complications. Multivariate analysis identified ICUD as a protective factor against late-stage adverse events (OR; 0.68). This finding aligns with a systematic review by Katayama et al. [13], suggesting that avoiding laparotomy and minimizing bowel exposure reduces the risk of long-term complications. Furthermore, reduced manipulation of the mesentery in ICUD is crucial in preserving the blood supply to the distal ureters and ileal segment, lowering the rates of ureteroileal strictures. Regarding the laparoscopic series, Hatakeyama [14] supports the notion that maintaining a closed environment preserves peritoneal physiology, which is crucial for long-term functional recovery.

Postoperative ileus (POI) remains a common reason for prolonged hospital stay after radical cystectomy. Proponents of ICUD have long argued that by avoiding the “cooling” and “drying” of the bowel that occurs during ECUD, the return of gastrointestinal motility should be faster. Tanneru et al. [15] and Katayama et al. [13] reported a trend toward faster return of bowel function and shorter LOS with the ICUD approach. Our data indicated a favorable recovery profile for patients with ICUD.

As the population ages, a significant number of cystectomy candidates are aged > 65 years. Recently, Cao et al. [16] and Kossenas et al. [17] addressed the ICUD vs. ECUD debate in the elderly population. Their findings suggest that although elderly patients are more susceptible to the stressors of surgery, they may benefit more from the reduced surgical trauma of ICUD. Our findings extend this observation by demonstrating that ICUD is feasible and superior regarding long-term safety in the elderly. Although ICUD was associated with a significantly longer operative time across all age groups (*p* < 0.001), it did not translate into increased perioperative morbidities. Conversely, patients aged < 80 years in the ICUD group showed significantly lower late complication rates than those in the ECUD group (*p* < 0.05). Even in the most elderly cohort (≥ 80 years), ICUD remained safe, with no significant differences in major complications or hospital stay compared to ECUD. Thus, the minimal invasiveness of ICUD, which avoids even a small laparotomy and maintains a closed intra-abdominal environment, provides a critical advantage for frail patients with limited physiological reserves, reducing delayed surgical sequelae.

Given that ileal conduit and orthotopic neobladder differ in technical complexity and reconstruction time, we additionally examined whether the impact of ICUD varied by diversion type. The longer operative time associated with ICUD was observed in both subgroups, suggesting that the time penalty of an entirely intracorporeal approach is consistent across diversion types. In contrast, the effect of ICUD on blood loss differed according to reconstruction type. ICUD was associated with higher estimated blood loss in the ileal conduit subgroup, but with significantly lower blood loss in the neobladder subgroup. This finding suggests that avoiding a mini-laparotomy may be particularly advantageous during more complex reconstruction.

The lower rate of late postoperative complications observed in the overall cohort appeared to be driven mainly by the ileal conduit subgroup, in which ICUD remained independently associated with reduced late complications after adjustment. In the neobladder subgroup, late complication rates were comparable between ICUD and ECUD. This finding should be interpreted cautiously, given the relatively small number of ICUD neobladder cases (*n* = 44), which limits statistical power. Overall, these results suggest that the clinical impact of ICUD is not uniform across diversion types. Therefore, ileal conduit and neobladder outcomes should be considered separately when evaluating the relative benefits of intracorporeal and extracorporeal urinary diversion.

A primary concern with any new surgical technique is whether it compromises the oncological efficacy. Our results showed no significant differences in positive surgical margin (rates or lymph node yield between ICUD and ECUD, which is consistent with the International Robotic Cystectomy Consortium database [18]. Furthermore, in considering the type of diversion, such as the orthotopic neobladder, studies by Mistretta et al. [19] and Yan et al. [20] have shown that functional outcomes, including daytime and nighttime continence, are comparable, if not slightly better, with the ICUD owing to the superior visualization of the urethral-neobladder anastomosis. The KORARC data highlighted that ICUD is becoming the preferred approach in high-volume Republic of Korean centers. These findings are also consistent with previous comparative studies evaluating perioperative outcomes between ICUD and ECUD.

Despite the large sample size and multi-institutional nature of our study, several limitations must be acknowledged. The retrospective nature of the database introduced an inherent selection bias. Future research should focus on long-term quality of life metrics and cost-effectiveness. Randomized trials with long-term follow-ups will continue to refine our understanding of which patients will benefit the most from each approach.

## Conclusion

This multi-institutional study demonstrated that RARC with ICUD is a safe and effective alternative to ECUD. Although ICUD is associated with longer operative times, it offers a significant advantage in reducing late postoperative complications and improving gastrointestinal recovery. This benefit was most evident in the ileal conduit subgroup, while ICUD remained safe and was associated with lower blood loss in neobladder cases. As surgical teams move beyond the learning curve, ICUD may become the standard of care for RARC.

## Supplementary Information

Below is the link to the electronic supplementary material.

Supplementary Material 1

Supplementary Material 2

Supplementary Material 3

## Data Availability

The data supporting the findings of this study are available from the corresponding author upon reasonable request.
