# Supplementary material for: Perioperative outcomes of intracorporeal versus extracorporeal urinary diversion following robot-assisted radical cystectomy in multicenter KORARC database
Source: World J Urol. 2026 Oct 27;44(1):763. doi: 10.1007/s00345-026-06550-7 (PMC13713994; doi:10.1007/s00345-026-06550-7)
Supplement: Supplementary file 1 [file 345_2026_6550_MOESM3_ESM.docx]

**Supplementary Table S1. Baseline characteristics and perioperative outcomes in the ileal conduit subgroup (n=384)**

|  | **ICUD (n=130)** | **ECUD (n=254)** | **p-value** |
| --- | --- | --- | --- |
| Age (years) | 61.2 ± 10.3 | 61.2 ± 10.4 | 0.988 |
| BMI (kg/m²) | 24.5 ± 3.5 | 24.5 ± 3.1 | 0.930 |
| Total operative time (min) | 514.6 ± 118.2 | 453.8 ± 139.4 | <0.001 |
| Estimated blood loss (mL) | 647.6 ± 521.3 | 526.5 ± 379.5 | 0.034 |
| Hospital stay (days) | 22.7 ± 16.6 | 22.7 ± 10.9 | 0.959 |
| Intraoperative transfusion | 15 (11.5%) | 29 (11.4%) | 1.000 |
| Any complication | 73 (56.2%) | 155 (61.0%) | 0.381 |
| - Early (≤30 days) | 22 (16.9%) | 46 (18.1%) | 0.888 |
| - Late (30–90 days) | 40 (30.8%) | 105 (41.3%) | 0.046 |
| Major complication (Clavien ≥3) | 34 (26.2%) | 87 (34.3%) | 0.131 |

**Supplementary Table S2. Baseline characteristics and perioperative outcomes in the orthotopic neobladder subgroup (n=272)**

|  | **ICUD (n=44)** | **ECUD (n=228)** | **p-value** |
| --- | --- | --- | --- |
| Age (years) | 70.5 ± 8.4 | 68.5 ± 10.0 | 0.169 |
| BMI (kg/m²) | 24.9 ± 3.7 | 24.0 ± 3.0 | 0.130 |
| Total operative time (min) | 470.6 ± 102.8 | 405.6 ± 108.2 | <0.001 |
| Estimated blood loss (mL) | 375.8 ± 248.6 | 518.6 ± 385.2 | 0.003 |
| Hospital stay (days) | 25.3 ± 55.4 | 16.0 ± 8.5 | 0.268 |
| Intraoperative transfusion | 5 (11.4%) | 55 (24.1%) | 0.074 |
| Any complication | 24 (54.5%) | 142 (62.3%) | 0.399 |
| - Early (≤30 days) | 5 (11.4%) | 37 (16.2%) | 0.501 |
| - Late (30–90 days) | 15 (34.1%) | 89 (39.0%) | 0.613 |
| Major complication (Clavien ≥3) | 12 (27.3%) | 69 (30.3%) | 0.857 |

**Supplementary Table S3. Univariate and multivariate logistic regression analysis for late postoperative complications in the ileal conduit subgroup**

|  | **Univariate OR (95% CI)** | **p-value** | **Multivariate OR (95% CI)** | **p-value** |
| --- | --- | --- | --- | --- |
| Age (years) | 1.02 (1.00-1.04) | 0.138 | 1.01 (0.99-1.03) | 0.273 |
| BMI (kg/m²) | 0.97 (0.91-1.04) | 0.431 | 0.98 (0.92-1.05) | 0.583 |
| ASA score (≥3 vs. 1-2) | 1.46 (0.52-4.13) | 0.470 | 1.18 (0.39-3.58) | 0.764 |
| Pathological T stage (≥pT3 vs. ≤pT2) | 1.16 (0.74-1.81) | 0.519 | 0.93 (0.56-1.57) | 0.797 |
| Pathological N stage (Positive vs. Negative) | 1.61 (0.97-2.67) | 0.067 | 1.71 (0.95-3.08) | 0.071 |
| Diversion approach (ICUD vs. ECUD) | 0.63 (0.40-0.99) | 0.044 | 0.60 (0.38-0.95) | 0.029 |

**Supplementary Table S4. Univariate and multivariate logistic regression analysis for late postoperative complications in the orthotopic neobladder subgroup**

|  | **Univariate OR (95% CI)** | **p-value** | **Multivariate OR (95% CI)** | **p-value** |
| --- | --- | --- | --- | --- |
| Age (years) | 1.01 (0.98-1.03) | 0.627 | 1.02 (0.99-1.04) | 0.272 |
| BMI (kg/m²) | 0.95 (0.88-1.03) | 0.254 | 0.97 (0.89-1.05) | 0.454 |
| ASA score (≥3 vs. 1-2) | 0.54 (0.24-1.21) | 0.136 | 0.54 (0.23-1.29) | 0.167 |
| Pathological T stage (≥pT3 vs. ≤pT2) | 0.96 (0.58-1.57) | 0.859 | 0.80 (0.46-1.40) | 0.440 |
| Pathological N stage (Positive vs. Negative) | 1.53 (0.88-2.67) | 0.136 | 1.52 (0.82-2.82) | 0.180 |
| Diversion approach (ICUD vs. ECUD) | 0.81 (0.41-1.59) | 0.537 | 0.76 (0.37-1.56) | 0.462 |
